# Environmental Stress and Plants

**DOI:** 10.3390/ijms23105416

**Published:** 2022-05-12

**Authors:** Lavinia Mareri, Luigi Parrotta, Giampiero Cai

**Affiliations:** 1 Department of Life Sciences, University of Siena, Via P.A. Mattioli 4, 53100 Siena, Italy; lavinia.mareri@unisi.it (L.M.); cai@unisi.it (G.C.); 2 Department of Biological, Geological and Environmental Sciences, University of Bologna, Via Irnerio 42, 40126 Bologna, Italy; 3 Interdepartmental Centre for Agri-Food Industrial Research, University of Bologna, Via Quinto Bucci 336, 47521 Cesena, Italy

**Keywords:** abiotic and biotic stresses, plant response, plant adaptation, multidisciplinary approaches

## Abstract

Land plants are constantly subjected to multiple unfavorable or even adverse environmental conditions. Among them, abiotic stresses (such as salt, drought, heat, cold, heavy metals, ozone, UV radiation, and nutrient deficiencies) have detrimental effects on plant growth and productivity and are increasingly important considering the direct or indirect effects of climate change. Plants respond in many ways to abiotic stresses, from gene expression to physiology, from plant architecture to primary, and secondary metabolism. These complex changes allow plants to tolerate and/or adapt to adverse conditions. The complexity of plant response can be further influenced by the duration and intensity of stress, the plant genotype, the combination of different stresses, the exposed tissue and cell type, and the developmental stage at which plants perceive the stress. It is therefore important to understand more about how plants perceive stress conditions and how they respond and adapt (both in natural and anthropogenic environments). These concepts were the basis of the Special Issue that *International Journal of Molecular Sciences* expressly addressed to the relationship between environmental stresses and plants and that resulted in the publication of 5 reviews and 38 original research articles. The large participation of several authors and the good number of contributions testifies to the considerable interest that the topic currently receives in the plant science community, especially in the light of the foreseeable climate changes. Here, we briefly summarize the contributions included in the Special Issue, both original articles categorized by stress type and reviews that discuss more comprehensive responses to various stresses.

## 1. Temperature Stress

The most prominent feature of climate change is temperature fluctuation. Several authors have studied genetic and molecular aspects related to plant response to temperature stresses. Park et al. [1] used QTL mapping to identify candidate genes for tolerance during the rice booting phase; among them, the *OsBHT* gene (encoding an Hsps-p23-like calcyclin-binding protein) showed a different expression level between a tolerant line (CNDH75) and a susceptible line (CNDH11). Therefore, *OsBHT* might be effectively used for the development of heat tolerance in rice. In contrast, Aleynova et al. [2] focused on calmodulin-like proteins (CMLs), which act as protein sensors in response to environmental cues. The authors reported that the four alternatively spliced mRNAs from the grapevine *CML21* gene are highly induced by low temperature with two forms (*VaCML21v1* and *VaCML21v2*) also modulated by other abiotic stresses (desiccation, heat, high salt, and mannitol). Interestingly, the heterologous expression of *VaCML21v2* and *VaCML21v4* in Arabidopsis modulates the expression of cold stress response marker genes from the AtDREB, AtRD, and AtKIN families. A further study by Kumar et al. [3] used a combination of computational and laboratory approaches to obtain more information about the PIN-FORMED (PIN) gene family in common wheat. The researchers assessed the role of the PIN gene family (responsible for PIN-mediated auxin transport) in various developmental processes and different biotic and abiotic stress conditions. This work provides valuable information about the *TaPIN* gene family and their functions in various plant developmental processes, as well as in response to hormones and stress. Zhang et al. [4] studied the expression of cytosolic glucose-6-phosphate dehydrogenase (CY) (*G6PDH*), which plays an important role in plant stress responses in strawberries. The authors reported that *FaG6PDH-CY* is a highly expressed gene in the investigated tissues and positively regulates cold tolerance in strawberries. The results indicate that *FaG6PDH-CY* enhances the ROS-scavenging capacity of antioxidant enzymes, thereby suppressing excessive accumulation of ROS and relieving oxidative damage. A further study by Huong et al. [5] focuses on an epigenetic mechanism based on RNA methylation and demethylation. Specifically, the authors analyzed the function of a potential eraser protein, ALKBH6 (At4g20350), during seed germination and the growth of Arabidopsis seedlings under abiotic stresses. The results indicate that ALKBH6 (a potential RNA demethylase) plays important roles in the survival of Arabidopsis thaliana under high temperature regimes. Considering climate change, many studies are attempting to identify feasible mitigation strategies. The application of both exogenous melatonin (a hormone that enhances plant response to abiotic stresses) and elevated CO_2_ concentration (e[CO_2_]) is attracting interest. Zhou et al. [6] studied the effect of elevated CO_2_ concentrations combined with melatonin in tomato plants under drought and cold stress. While the photosynthesis rate (PN) is decreased by water deficit, it is restored after recovery from drought but remains low after subsequent cold stress. In contrast, melatonin enhances PN during recovery from drought and cold stress by increasing biomass accumulation in tomato under e[CO_2_]. Another strategy is the application of priming, which consists of a gradual increase in temperature or a brief pre-exposure to a slightly supra-optimal but nonlethal temperature. Mareri et al. [7] studied the effects of priming on pollen grain function in tobacco. The results showed that pre-exposure to sub-lethal temperatures (30 °C) positively improves pollen performance by altering its metabolism. This may have considerable impact, especially from the perspective of breeding strategies to improve crop species. Heat priming was also tested by Bhardwaj et al. [8] on lentil (*Lens culinaris* Medik) seeds along with foliar treatment with γ-aminobutyric acid (GABA), separately or in combination. Analyses of both heat-stress-tolerant and heat-stress-susceptible genotypes showed that germination and pollen grain viability, stigma receptivity, and ovule viability were significantly improved under the combined treatment, which was more effective in heat-sensitive genotypes.

## 2. Drought Stress

One of the negative effects of climate change is soil water deficit, which results in drought stress. Many studies applied a molecular approach to identify involved mechanisms underlying drought tolerance. Waititu and colleagues [9] performed transcriptomic and physiological analyses on seedlings of drought-tolerant (CML69) and drought-sensitive (LX9801) maize (*Zea mays* L.) inbred lines. RNA-seq analysis identified differentially expressed genes (DEGs) expressed by the CML69 tolerant line in association with cytoskeleton, cell wall, metabolism, transport, osmotic regulation, drought prevention, ROS scavengers, and defense; DEGs identified in the susceptible line involved photosynthesis, histones, and carbon fixation pathways. These results provide a basis for the molecular networks mediating drought stress tolerance of maize seedlings. Drought stress was also studied in alfalfa (*Medicago sativa*) by investigating the role of Squamosa Promoter Binding Protein-Like 9 (SPL9, target of miR156) in drought tolerance. Authors reported that SPL9-RNAi plants had phenotypic changes compared to the control including a reduction in stem thickness, plant height, and less leaf senescence and increased relative water content under drought stress. Interestingly, SPL9-RNAi plants accumulated more antistress anthocyanin than Wild Type (WT), suggesting that MsSPL9 contributes to drought tolerance by regulating anthocyanin biosynthesis [10]. Drought causes excessive flower abscission in yellow lupine, leading to yield loss and economic consequences in agriculture. Florkiewicz and colleagues [11] tested the hypothesis that soil water limitation disturbs auxin balance in the abscission zone leading to flower drop. Drought results in the accumulation of proline in the abscission zone. In addition, the cell wall changes in response to drought by reorganizing methylated homogalacturonans in the abscission zone and upregulating pectin methylesterase and polygalacturonase. In a water-limiting environment, abscisic acid (ABA) is involved in drought tolerance. Pyrabactin resistance-like (PYL) proteins act as ABA receptors; Usman and coworkers [12] mutagenized rice plants with CRISPR/Cas9 in the OsPYL9 gene. The mutant lines showed ABA accumulation, increased antioxidant activities and chlorophyll content, but lower levels of malondialdehyde, stomatal conductance, and transpiration rate under stress conditions. The results indicate that OsPYL9 mutants have enhanced drought tolerance and yield. The relationship between microbiome and roots is important for plants, but the role of the endophytic microbiome of spelt (*Triticum aestivum* ssp. spelta L.) roots under drought conditions is poorly understood. Ratajczak et al. [13] found that inoculating the tolerant ‘Badenstern’ variety with the mycorrhizal fungus *R. irregularis* contributed to improved growth performance. The inoculation of less tolerant varieties (such as “Zollernspelz”) also produces positive effects on the roots under stress. The study provides evidence that arbuscular mycorrhizal fungi are beneficial for the growth of spelt varieties under drought conditions. The inoculation with arbuscular mycorrhizal fungi (AMF) enhances the efficiency of Photosystem II (PSII) and improves root growth under drought conditions (confirmed by enhanced aboveground biomass, root dry weight and length). Recent literature proposes a key role for UDP-glycosyltransferases (UGT) in plant development and environmental responses. In Arabidopsis, UDP-glucosyltransferase 74E2 (*AtUGT74E2*) transfers glucose to indole-3-butyric acid (IBA), and it is involved in stress response. Wang et al. [14] found that overexpression of *AtUGT74E2* in rice improves seed germination in the presence of IBA and abscisic acid (ABA) as well as saline and drought stresses. The distinct roles of *UGT74E2* indicate different regulatory networks between Arabidopsis and rice. Rice is also used in the study of Wytynck et al. [15] to investigate the role of ribosome-inactivating proteins (RIPs). RIPs are enzymes capable of irreversible modification of ribosomal RNAs. Some experiments have suggested that cereal RIPs may play a role in abiotic stress tolerance and in plant defense. In this work, transgenic rice lines overexpressing RIPs have been studied for the effects on seedling development in response to drought, salt, abscisic acid, and methyl gelsominate treatment. The results suggest that RIPs may influence methyl jasmonate-mediated stress responses. Like drought, another abiotic stress that negatively affects annual wheat production is waterlogging, which significantly reduces the rate of oxygen diffusion in the soil. Koramutla et al. [16] studied the role of salicylic acid (SA) in regulating morphological adaptive responses of wheat to waterlogging. Treatment of water-logged wheat plants with SA promotes the formation of axial roots and superficial adventitious roots but inhibits their elongation, thereby generating a shallow root system. The results highlight that SA promotes the development of axial and superficial adventitious roots in water-logged wheat plants in an ethylene-independent way.

## 3. Salt and Osmotic Stress

Excess salinity is a major environmental threat to crops because high salt concentration in soil alters plant performance by causing metabolic damage, ion toxicity, and leading to secondary oxidative stress and osmotic stress. As salt stress is directly linked to osmotic stresses, they will be presented together. Salt stress induces a deep reprogramming of gene expression that is the basis for an efficient salt stress response. Wang et al. [17] focus their attention to sugar beet BvbHLH93, a transcriptional factor belonging to the basic/helix–loop–helix (bHLH) family that plays an important role for plant growth, development, and stress responses. The authors report that BvbHLH93 positively regulates the expression of genes for SOD (superoxide dismutase) and POD (peroxidase) genes, encoding for enzymes involved in ROS-detoxifying pathways. In addition to this, plants overexpressing BvBHLH93 exhibit low expression levels of RbohD and RbohF, both involved ROS production. Thus, it is likely that BvbHLH93 plays a key role in improving salt stress tolerance by increasing the content of antioxidant enzymes and reducing the levels of those involved in ROS production. An additional family of transcriptional factors involved in both salt and osmotic stress, as well as many other stresses, is the DREB (Dehydration-responsive element-binding) transcription factor that belongs to the AP2/EREBP family. Wang and colleagues [18] studied the expression of three members of DREB family in wheat AK58, and they also analyzed the profile of promoter methylation under osmotic stress. The results highlighted that the expression of *DREB2*, *DREB6*, and *Wdreb2* was tissue-specific and was highest in leaves, and there were expression changes with increasing osmotic stress. In addition, considering the status of promoter methylation and its influence on gene expression, authors suggested different functions of *DREB2*, *DREB6*, and *Wdreb2* in response to osmotic stress. The influence of salt stress on the expression of genes encoding the mitochondrial and cytosolic forms of aconitase and fumarase was studied in maize (*Zea mays* L.) considering promoter methylation as a regulatory mechanism of gene expression. The authors reported an enhanced expression of genes for the mitochondrial forms of aconitase (*Aco1* and *Aco2*) and fumarase (Fum1) corresponding to increased transcription of *ZmCOI6.1*, a general stress response marker gene. This suggests that the activation of the aconitase and fumarase genes might be controlled in the same way [19]. The work of Wang and colleagues [14] focused on the role of the UDP-glucosyltransferase 74E2 (AtUGT74E2), which transfers glucose to indole-3-butyric acid (IBA) and is involved in regulating plant architecture and stress responses. The overexpression of *AtUGT74E2* in rice enhances seed germination and AtUGT74E2-overexpressing lines had lower contents of free IBA and AB compared to WT. Genes involved in both the auxin (i.e., *OsARF* and *OsGH3*) and ABA signaling pathways (i.e., *OsABI3* and *OsABI5*) were downregulated in germinating seeds of UGT74E2-overexpressing lines. Seedlings were less tolerant to drought and salt stresses such that stress tolerance could be attributed to IBA and ABA level alterations, as well as modulation of the auxin/ABA signaling pathways by UGT74E2. Soil salinization has also been studied in cotton. The results of the work by Ma et al. [20] provide the basis for a detailed characterization of the regulation of salt tolerance-related Na^+^/H^+^ antiporter (NHX) genes in cotton, especially the endosomal *GhNHX4A*. The data may be useful for selecting appropriate candidate genes for breeding new salt-tolerant cotton varieties. Drought and soil salinity lead to osmotic stress and are one of the main abiotic stresses affecting agricultural production. The study by Jiroutova and coworkers [21] focused on antioxidant activity, osmotic accumulation, and abscisic acid content in apples and cherries grown in soils with different levels of polyethylene glycol PEG-6000. Genotypes responded differently to osmotic stress with reduced leaf water content (RWC) and increased activities of antioxidant enzymes, proline, sugars, and ABA content. Cherry cultivars showed a lower decrease in RWC and enzyme activities, but higher proline content than apple cultivars. The work of Wang et al. [22] focused on dehydrins (DHNs) in *Larix kaempferi* that improve plant tolerance to abiotic stress. The results indicated that four LkDHNs help plants to survive stress by protecting DNA. The four LkDHNs have similar roles in osmotic stress response and helped adaptation to the dry and cold winter of northern China. Sugar beet is an important salt-tolerant crop, but the basis of this tolerance is unknown. In the work of Li et al. [23], the authors used RNA-seq whole-transcriptome and degradome sequencing in response to salt stress to discover differentially expressed mRNAs (DEs), microRNAs (miRNAs), long noncoding RNAs (lncRNAs), and circular RNAs (circRNAs) in leaves and roots. This study represents for the first time a full transcriptomic analysis of sugar beet under salt stress that involves a potential competitive endogenous RNA (ceRNA) network, thus providing a basis to study the potential functions of lncRNAs/circRNAs. Orzechowska and colleagues [24] used a nondestructive thermal imaging method to study the stomatal response of *Arabidopsis thaliana* treated with salt and excessive light. The initial plant response associated with stomatal opening shows an exponential increase in temperature kinetics. Salt-induced alteration in stomata causes a reduction in stomatal conductance and transpiration rate, in turn causing an increase in rosette temperature. This study demonstrates that thermal imaging is sensitive and useful for analyzing stomatal opening under dynamic environmental conditions.

## 4. Ozone, UV and Light Stresses

Ultraviolet (UV) radiation, especially UV-B, has long been considered a stressor for plants, causing DNA, protein, and membrane damage. One of the strategies adopted by plants to counteract UV stress is the synthesis of antioxidant molecules (e.g., phenolic and flavonoid compounds) as well as UV-B screening molecules. In the study by Yoon et al. [25], authors investigated the spatial interception of UV-B radiation of kale (*Brassica oleracea* L. var. Acephala) grown under supplemental UV-B LED using ray-tracing simulation using a high-resolution portable 3D scanner and leaf optical properties. UV-B-induced phenolic compounds and flavonoids accumulated largely, and UV-B was more intercepted in younger leaves. The effect of the UV-B intercept on the flavonoid content was substantially higher than leaf age. Overall, the study paves the way to explore the physical and physiological basis of the intraindividual distribution of phenolic compounds. The study of Wójtowicz and co-workers [26] focused on a mutation, namely, ch1, that affects chlorophyllide an oxygenase (CAO), the enzyme responsible for chlorophyll *b* synthesis. The authors aimed to understand the strategy for compensation mechanism of the photosynthetic apparatus during low chlorophyll *b* content by characterizing and comparing the performance and spectral properties of the photosynthetic apparatus related to the lipid and protein composition in four selected Arabidopsis ch1 mutants and two Arabidopsis ecotypes. The exposure of mutants with lower chlorophyll *b* content to short-term and long-term low-light stress enabled a shift in the structure of both PSI and PSII via spectral analysis and thylakoid composition studies. Both ecotypes, Col-1 and Ler-0, reacted to high-light conditions in a way resembling the response of ch1 mutants to normal conditions. The authors suggested how the conversion of chlorophyll *a* to *b* might be regulated depending on the light stress conditions. 

## 5. Nutrient Stress

Abiotic stress and soil nutrient limitation are environmental conditions that reduce plant growth, productivity, and quality. In natural and agricultural ecosystems, one of the most common soil-related abiotic stress is low phosphorus (P) availability, which limits crop productivity in more than 70% of globally available arable land. To overcome the low availability of inorganic P in the soil, the application of large amounts of fertilizers is the main strategy to maintain crop yields. Although the molecular mechanisms of the low-P stress response have been studied in detail, the epigenetic regulatory mechanisms remain unknown. Chu et al. [27] evaluated changes in DNA methylation, gene expression, and siRNA abundance in response to low-P stress in two soybean genotypes with different P efficiencies. DNA methylation levels were higher under low-P stress in both genotypes, and transcriptional alterations in some genes were found to be associated with changes in methylation. A low availability of P is also a limiting factor for potatoes. P can become toxic when accumulated at high concentrations (500 µM). In the study by Chea and colleagues [28], plant morphology, mineral allocation, and metabolites were assessed under P deficiency and toxicity; the study also evaluated the ability of rhizobacteria to enhance plant biomass and P uptake. A reduction in plant height and biomass under P deficiency was observed, along with altered mineral concentration and allocation. The stress induced by P deficiency and toxicity was evident by the accumulation of proline. Hornyák and colleagues [29] studied nutritional stress in vitro and in planta, analyzing several embryological (e.g., developed ovules, embryo sacs, and pollen viability) and yield parameters. Flowers grown in vitro with severely reduced nutrient content showed dramatic degeneration of embryo sacs. In planta, reducing flower competition was found to be the most promising treatment to improve yield by increasing the frequency of developed embryo sacs and the average number of mature seeds. These effects could result from increased production of SA and jasmonic acid (JA) that promote more effective pollinator attraction. High bicarbonate concentrations in calcareous soils with high pH affect crop performance (e.g., Fe deficiency). The ability to mobilize poorly soluble Fe is key to tolerance. In the work of Pérez-Martín et al. [30], a comparative transcriptomic analysis performed on two *Arabidopsis thaliana* genotypes (carbonate-tolerant and -susceptible) revealed that bicarbonate rapidly induces Fe deficiency-related genes in the susceptible genotype. In contrast, the tolerant line showed a differential expression of gene for receptor-protein-like, jasmonate, and salicylate pathways; sulfur starvation; and starch degradation, suggesting that carbonate-tolerant plants do not sense Fe deficiency rapidly. Potassium (K^+^) is an essential macronutrient that plays crucial roles in plant growth, development, and stress response. To comprehend the responses of cotton to K^+^ deficiency, Yang and coworkers [31] analyzed the root transcriptome after low-K^+^ treatment (0.03 mM in hydroponic cultivation). The results highlighted several genes associated with tolerance to low K^+^ that need to be further identified and characterized. Sub-optimal growing conditions can have important effects on plants and increase the sustainable use of non-renewable inputs. Miras-Moreno and colleagues [32] investigated the impact of sub-optimal availability of macrocations and different light intensities in two varieties of lettuce that differ in the accumulation of secondary metabolites. Several stress-related metabolites (such as polyamines) were altered by treatments suggesting that effects on sustainable low-input agricultural systems should be evaluated by considering positive and disadvantageous metabolic effects in addition to yield and socio-economic parameters.

## 6. Heavy Metal Stress

Exposure to heavy metals impairs morphological, physiological, biochemical, and molecular processes in plants. Pb and Cd in the environment severely affects plant growth and yield. In contrast, plants acquire Zn from soil for their vital functions. Shafiq and co-authors [33] report that Zn facilitates the accumulation and transport of Pb and Cd in the aerial parts of maize plants. In addition, the interaction of Zn, Pb, and Cd interferes with the uptake and translocation of other divalent metals. This study highlights how DNA methylation and histone acetylation affect metal stress tolerance through Zn transporters and alerts against the overuse of Zn fertilizers in metal-contaminated soils. Cerium dioxide (CeO_2_) nanoparticles are pollutants of emerging concern as they are rarely immobilized in the environment. In the study of Skiba et al. [34], CeO_2_ nanoparticles (CNPs) were proved to affect metals uptake. In particular, a decrease in Cu, Zn, Mn, Fe, and Mg is found in the roots while a reversed process was observed for Ca. This study is well suited for investigating the interactions induced by CNPs, which affect photosynthesis-related parameters in pea. Acyl activating enzyme 3 (AAE3) was identified as being involved in the acetylation pathway of oxalate degradation, which regulates the responses to biotic and abiotic stresses in various higher plants. Xian and co-workers [35] investigated the role of *Glycine soja* AAE3 (*GsAAE3*) in Cd and Al sensitivity. *GsAAE3* overexpression increases Cd and Al tolerances in A. thaliana and soybean hairy roots, which is associated with a decrease in oxalate accumulation. Taken together, the data provide evidence that the *GsAAE3*-encoded protein plays an important role in coping with Cd and Al stresses. The presence of Al can be very toxic, especially in acidic soils. Mechanistically elucidating a plant’s response to Al stress is critical to mitigating this stress and improving the quality of plants. To identify the genes involved in sugarcane response to Al stress, Rosa-Santos and colleagues [36] generated 372 million paired-end RNA sequencing reads from the roots of CTC-2 and RB855453, which are two contrasting cultivars. The majority of the genes were upregulated in the CTC-2 (tolerant cultivar) and downregulated in RB855453 (sensitive cultivar). The results and conclusions of this study represent a starting point for future genetic and genomic studies of sugarcane. The transcriptome analysis shows that sugarcane tolerance to Al may be explained by an efficient detoxification mechanism combined with lateral root formation and activation of redox enzymes. Plants growing on heavy metal-polluted soils show toxicity symptoms, such as chlorosis and growth reduction, and undergo oxidative stress due to the formation of ROS. Plants overcome oxidative stress by producing a wide range of antioxidant molecules, such as polyphenols and flavonoids. The aim of Salinitro et al. [37] was to study the accumulation of these molecules in response to increasing concentrations of Cd, Cr, Cu, Ni, Pb, and Zn and to assess whether they can be used as a tool in assessing metal-related stress in *Polygonum aviculare* and *Senecio vulgaris*. This research demonstrated that 82% of the samples showed a good correlation between the level of polyphenols, flavonoids, and antioxidant activity and the metal concentration in plant shoots, confirming that the metal stress level and production of phenolic compounds having antioxidant activity were strictly connected.

## 7. Hypoxia Stress

In response to hypoxia under flooding, plants switch from aerobic respiration to anaerobic fermentation, with accumulation of the end product ethanol. Autophagy-deficient *Arabidopsis thaliana* mutants show increased sensitivity to ethanol treatment, indicating that ethanol is involved in regulating the autophagy-mediated response to hypoxia. Yuan et al. [38] used a transcriptomic analysis by which they identified 3909 genes in *A. thaliana* seedlings that were differentially expressed in response to ethanol treatment, including 2487 upregulated and 1422 downregulated. The data confirmed that ethanol treatment significantly upregulated genes involved in autophagy and ROS detoxification.

## 8. Reviews

Adverse environmental conditions are among the leading causes of declining crop yields worldwide. Many researchers are working on this topic, investigating and testing multiple solutions to achieve enhanced crop resilience to the harsh environment. The review of Hasanuzzaman et al. [39] discussed the physicochemical basis of ROS production, cellular compartment-specific ROS generation pathways, and their possible distressing effects. Moreover, the authors highlight the function of the antioxidant defense system for detoxification and homeostasis of ROS in light of the latest research. In addition, stress signal sensing is a crucial step for appropriate response and plant survival. As important signaling modules in eukaryotes, plant mitogen-activated protein kinase (MAPK) cascades play a key role in regulating responses to environmental stresses, such as high salinity, drought, extreme temperature, and insect and pathogen infections [40]. The survival strategies of plants adaptation to flooding stress at the morphological, physiological, and anatomical scale systemically, such as the formation of adventitious roots (ARs), aerenchyma, and radial O_2_ loss (ROL) barriers were reported in the review of Jia et al. [41]. The review of Hasan et al. [42] explores the effects of O_3_ on stomatal regulation through guard cell signaling by phytohormones. In this review, the authors updated the existing knowledge by considering several physiological mechanisms related to ozone-induced stomatal regulation. The information will deepen our understanding of the molecular pathways associated with the O_3_ stress response, specifically how it affects stomatal regulation, MAPK activity, and phytohormone signaling. Abiotic stresses disrupt K^+^ translocation and homeostasis; the review by Monder and colleagues [43] discusses the main negative consequences of the current climate on grape quality and thus wine quality. The essential electrical and osmotic functions of K^+^ are presented, which are intimately dependent on transport systems, membrane energetics, and cellular K^+^ homeostasis. The new knowledge will help the application of stress responsive determinants and in engineering plants with higher stress tolerance.

In conclusion, abiotic stresses will unfortunately persist as a constant challenge to the natural environment and agriculture. Diverse but mostly adverse conditions affect plant and crop productivity, reducing food availability and increasing the cost of production. The current limited availability of arable land, the growing world population, and decreasing water resources are challenges to produce more even in a scenario of impending climate change. Increasing knowledge of plant biology and crop improvement are strategic milestones to better understand abiotic stress responses, to identify stress protection networks, and to design environmentally stable crops that are more productive and resilient to environmental changes. The papers in this Special Issue highlighted several aspects involved in abiotic stress responses and attest to the growing interest of the scientific community in identifying valuable solutions.

## References

[1] Park J.-R., Yang W.-T., Kim D.-H., Kim K.-M. (2020). Identification of a Novel Gene, *Osbht*, in Response to High Temperature Tolerance at Booting Stage in Rice. Int. J. Mol. Sci..

[2] Aleynova O.A., Kiselev K.V., Ogneva Z.V., Dubrovina A.S. (2020). The Grapevine Calmodulin-Like Protein Gene *CML21* Is Regulated by Alternative Splicing and Involved in Abiotic Stress Response. Int. J. Mol. Sci..

[3] Kumar M., Kherawat B.S., Dey P., Saha D., Singh A., Bhatia S.K., Ghodake G.S., Kadam A.A., Kim H.-U., Manorama (2021). Genome-Wide Identification and Characterization of PIN-FORMED (PIN) Gene Family Reveals Role in Developmental and Various Stress Conditions in *Triticum aestivum* L.. Int. J. Mol. Sci..

[4] Zhang Y., Luo M., Cheng L., Lin Y., Chen Q., Sun B., Gu X., Wang Y., Li M., Luo Y. (2020). Identification of the Cytosolic Glucose-6-Phosphate Dehydrogenase Gene from Strawberry Involved in Cold Stress Response. Int. J. Mol. Sci..

[5] Huong T.T., Ngoc L.N.T., Kang H. (2020). Functional Characterization of a Putative RNA Demethylase ALKBH6 in *Arabidopsis* Growth and Abiotic Stress Responses. Int. J. Mol. Sci..

[6] Zhou R., Wan H., Jiang F., Li X., Yu X., Rosenqvist E., Ottosen C.-O. (2020). The Alleviation of Photosynthetic Damage in Tomato under Drought and Cold Stress by High CO_2_ and Melatonin. Int. J. Mol. Sci..

[7] Mareri L., Faleri C., Aloisi I., Parrotta L., Del Duca S., Cai G. (2021). Insights into the Mechanisms of Heat Priming and Thermotolerance in Tobacco Pollen. Int. J. Mol. Sci..

[8] Bhardwaj A., Sita K., Sehgal A., Bhandari K., Kumar S., Prasad P.V.V., Jha U., Kumar J., Siddique K.H.M., Nayyar H. (2021). Heat Priming of Lentil (*Lens culinaris* Medik.) Seeds and Foliar Treatment with γ-Aminobutyric Acid (GABA), Confers Protection to Reproductive Function and Yield Traits under High-Temperature Stress Environments. Int. J. Mol. Sci..

[9] Waititu J.K., Zhang X., Chen T., Zhang C., Zhao Y., Wang H. (2021). Transcriptome Analysis of Tolerant and Susceptible Maize Genotypes Reveals Novel Insights about the Molecular Mechanisms Underlying Drought Responses in Leaves. Int. J. Mol. Sci..

[10] Hanly A., Karagiannis J., Lu Q.S.M., Tian L., Hannoufa A. (2020). Characterization of the Role of *SPL9* in Drought Stress Tolerance in *Medicago sativa*. Int. J. Mol. Sci..

[11] Florkiewicz A.B., Kućko A., Kapusta M., Burchardt S., Przywieczerski T., Czeszewska-Rosiak G., Wilmowicz E. (2020). Drought Disrupts Auxin Localization in Abscission Zone and Modifies Cell Wall Structure Leading to Flower Separation in Yellow Lupine. Int. J. Mol. Sci..

[12] Usman B., Nawaz G., Zhao N., Liao S., Liu Y., Li R. (2020). Precise Editing of the *OsPYL9* Gene by RNA-Guided Cas9 Nuclease Confers Enhanced Drought Tolerance and Grain Yield in Rice (*Oryza sativa* L.) by Regulating Circadian Rhythm and Abiotic Stress Responsive Proteins. Int. J. Mol. Sci..

[13] Ratajczak K., Sulewska H., Błaszczyk L., Basińska-Barczak A., Mikołajczak K., Salamon S., Szymańska G., Dryjański L. (2020). Growth and Photosynthetic Activity of Selected Spelt Varieties (*Triticum aestivum* ssp. spelta L.) Cultivated under Drought Conditions with Different Endophytic Core Microbiomes. Int. J. Mol. Sci..

[14] Wang T., Li P., Mu T., Dong G., Zheng C., Jin S., Chen T., Hou B., Li Y. (2020). Overexpression of *UGT74E2*, an Arabidopsis IBA Glycosyltransferase, Enhances Seed Germination and Modulates Stress Tolerance via ABA Signaling in Rice. Int. J. Mol. Sci..

[15] Wytynck P., Lambin J., Chen S., Demirel Asci S., Verbeke I., De Zaeytijd J., Subramanyam K., Van Damme E.J.M. (2021). Effect of RIP Overexpression on Abiotic Stress Tolerance and Development of Rice. Int. J. Mol. Sci..

[16] Koramutla M.K., Tuan P.A., Ayele B.T. (2022). Salicylic Acid Enhances Adventitious Root and Aerenchyma Formation in Wheat under Waterlogged Conditions. Int. J. Mol. Sci..

[17] Wang Y., Wang S., Tian Y., Wang Q., Chen S., Li H., Ma C., Li H. (2021). Functional Characterization of a Sugar Beet *BvbHLH93* Transcription Factor in Salt Stress Tolerance. Int. J. Mol. Sci..

[18] Wang H., Zhu Y., Yuan P., Song S., Dong T., Chen P., Duan Z., Jiang L., Lu L., Duan H. (2021). Response of Wheat DREB Transcription Factor to Osmotic Stress Based on DNA Methylation. Int. J. Mol. Sci..

[19] Eprintsev A.T., Fedorin D.N., Cherkasskikh M.V., Igamberdiev A.U. (2021). Effect of Salt Stress on the Expression and Promoter Methylation of the Genes Encoding the Mitochondrial and Cytosolic Forms of Aconitase and Fumarase in Maize. Int. J. Mol. Sci..

[20] Ma W., Ren Z., Zhou Y., Zhao J., Zhang F., Feng J., Liu W., Ma X. (2020). Genome-Wide Identification of the *Gossypium hirsutum* NHX Genes Reveals That the Endosomal-Type *GhNHX4A* Is Critical for the Salt Tolerance of Cotton. Int. J. Mol. Sci..

[21] Jiroutova P., Kovalikova Z., Toman J., Dobrovolna D., Andrys R. (2021). Complex Analysis of Antioxidant Activity, Abscisic Acid Level, and Accumulation of Osmotica in Apple and Cherry In Vitro Cultures under Osmotic Stress. Int. J. Mol. Sci..

[22] Wang X., Zhang M., Xie B., Jiang X., Gai Y. (2021). Functional Characteristics Analysis of Dehydrins in *Larix kaempferi* under Osmotic Stress. Int. J. Mol. Sci..

[23] Li J., Cui J., Dai C., Liu T., Cheng D., Luo C. (2021). Whole-Transcriptome RNA Sequencing Reveals the Global Molecular Responses and CeRNA Regulatory Network of mRNAs, lncRNAs, miRNAs and circRNAs in Response to Salt Stress in Sugar Beet (*Beta vulgaris*). Int. J. Mol. Sci..

[24] Orzechowska A., Trtílek M., Tokarz K.M., Szymańska R., Niewiadomska E., Rozpądek P., Wątor K. (2021). Thermal Analysis of Stomatal Response under Salinity and High Light. Int. J. Mol. Sci..

[25] Yoon H.I., Kim H.Y., Kim J., Oh M.-M., Son J.E. (2021). Quantitative Analysis of UV-B Radiation Interception in 3D Plant Structures and Intraindividual Distribution of Phenolic Contents. Int. J. Mol. Sci..

[26] Wójtowicz J., Jagielski A.K., Mostowska A., Gieczewska K.B. (2021). Compensation Mechanism of the Photosynthetic Apparatus in *Arabidopsis thaliana ch1* Mutants. Int. J. Mol. Sci..

[27] Chu S., Zhang X., Yu K., Lv L., Sun C., Liu X., Zhang J., Jiao Y., Zhang D. (2020). Genome-Wide Analysis Reveals Dynamic Epigenomic Differences in Soybean Response to Low-Phosphorus Stress. Int. J. Mol. Sci..

[28] Chea L., Pfeiffer B., Schneider D., Daniel R., Pawelzik E., Naumann M. (2021). Morphological and Metabolite Responses of Potatoes under Various Phosphorus Levels and Their Amelioration by Plant Growth-Promoting Rhizobacteria. Int. J. Mol. Sci..

[29] Hornyák M., Słomka A., Sychta K., Dziurka M., Kopeć P., Pastuszak J., Szczerba A., Płażek A. (2020). Reducing Flower Competition for Assimilates by Half Results in Higher Yield of *Fagopyrum esculentum*. Int. J. Mol. Sci..

[30] Pérez-Martín L., Busoms S., Tolrà R., Poschenrieder C. (2021). Transcriptomics Reveals Fast Changes in Salicylate and Jasmonate Signaling Pathways in Shoots of Carbonate-Tolerant *Arabidopsis thaliana* under Bicarbonate Exposure. Int. J. Mol. Sci..

[31] Yang D., Li F., Yi F., Eneji A.E., Tian X., Li Z. (2021). Transcriptome Analysis Unravels Key Factors Involved in Response to Potassium Deficiency and Feedback Regulation of K^+^ Uptake in Cotton Roots. Int. J. Mol. Sci..

[32] Miras-Moreno B., Corrado G., Zhang L., Senizza B., Righetti L., Bruni R., El-Nakhel C., Sifola M.I., Pannico A., Pascale S.D. (2020). The Metabolic Reprogramming Induced by Sub-Optimal Nutritional and Light Inputs in Soilless Cultivated Green and Red Butterhead Lettuce. Int. J. Mol. Sci..

[33] Shafiq S., Ali A., Sajjad Y., Zeb Q., Shahzad M., Khan A.R., Nazir R., Widemann E. (2020). The Interplay between Toxic and Essential Metals for Their Uptake and Translocation Is Likely Governed by DNA Methylation and Histone Deacetylation in Maize. Int. J. Mol. Sci..

[34] Skiba E., Pietrzak M., Gapińska M., Wolf W.M. (2020). Metal Homeostasis and Gas Exchange Dynamics in *Pisum sativum* L. Exposed to Cerium Oxide Nanoparticles. Int. J. Mol. Sci..

[35] Xian P., Cai Z., Cheng Y., Lin R., Lian T., Ma Q., Nian H. (2020). Wild Soybean Oxalyl-CoA Synthetase Degrades Oxalate and Affects the Tolerance to Cadmium and Aluminum Stresses. Int. J. Mol. Sci..

[36] Rosa-Santos T.M., Silva R.G.d., Kumar P., Kottapalli P., Crasto C., Kottapalli K.R., França S.C., Zingaretti S.M. (2020). Molecular Mechanisms Underlying Sugarcane Response to Aluminum Stress by RNA-Seq. Int. J. Mol. Sci..

[37] Salinitro M., Hoogerwerf S., Casolari S., Zappi A., Melucci D., Tassoni A. (2020). Production of Antioxidant Molecules in *Polygonum aviculare* (L.) and *Senecio vulgaris* (L.) under Metal Stress: A Possible Tool in the Evaluation of Plant Metal Tolerance. Int. J. Mol. Sci..

[38] Yuan L.-B., Chen L., Zhai N., Zhou Y., Zhao S.-S., Shi L.-L., Xiao S., Yu L.-J., Xie L.-J. (2020). The Anaerobic Product Ethanol Promotes Autophagy-Dependent Submergence Tolerance in *Arabidopsis*. Int. J. Mol. Sci..

[39] Hasanuzzaman M., Bhuyan M.H.M.B., Parvin K., Bhuiyan T.F., Anee T.I., Nahar K., Hossen M.S., Zulfiqar F., Alam M.M., Fujita M. (2020). Regulation of ROS Metabolism in Plants under Environmental Stress: A Review of Recent Experimental Evidence. Int. J. Mol. Sci..

[40] Lin L., Wu J., Jiang M., Wang Y. (2021). Plant Mitogen-Activated Protein Kinase Cascades in Environmental Stresses. Int. J. Mol. Sci..

[41] Jia W., Ma M., Chen J., Wu S. (2021). Plant Morphological, Physiological and Anatomical Adaption to Flooding Stress and the Underlying Molecular Mechanisms. Int. J. Mol. Sci..

[42] Hasan M.M., Rahman M.A., Skalicky M., Alabdallah N.M., Waseem M., Jahan M.S., Ahammed G.J., El-Mogy M.M., El-Yazied A.A., Ibrahim M.F.M. (2021). Ozone Induced Stomatal Regulations, MAPK and Phytohormone Signaling in Plants. Int. J. Mol. Sci..

[43] Monder H., Maillard M., Chérel I., Zimmermann S.D., Paris N., Cuéllar T., Gaillard I. (2021). Adjustment of K^+^ Fluxes and Grapevine Defense in the Face of Climate Change. Int. J. Mol. Sci..

